# Floquet edge states in germanene nanoribbons

**DOI:** 10.1038/srep31821

**Published:** 2016-08-23

**Authors:** M. Tahir, Q. Y. Zhang, U. Schwingenschlögl

**Affiliations:** 1 King Abdullah University of Science and Technology (KAUST), Physical Science and Engineering Division (PSE), Thuwal 23955-6900, Saudi Arabia

## Abstract

We theoretically demonstrate versatile electronic properties of germanene monolayers under circularly, linearly, and elliptically polarized light. We show for the high frequency regime that the edge states can be controlled by tuning the amplitude of the light and by applying a static electric field. For circularly polarized light the band gap in one valley is reduced and in the other enhanced, enabling single valley edge states. For linearly polarized light spin-split states are found for both valleys, being connected by time reversal symmetry. The effects of elliptically polarized light are similar to those of circularly polarized light. The transport properties of zigzag nanoribbons in the presence of disorder confirm a nontrivial nature of the edge states under circularly and elliptically polarized light.

External time-periodic perturbation by light is of great interest for studying quantum phase transitions^1^,^2^. Floquet bands were first observed in photonic crystals^3^ and have been verified experimentally for the surfaces of topological insulators^4^,^5^,^6^. For graphene the chiralities for different frequencies have been given in ref. 7 and a trivial band gap has been reported under high frequency linearly polarized light^8^. Light induced effects in silicene are limited to single Dirac cone states^9^. In contrast to graphene, silicene and germanene are subject to strong spin orbit coupling (SOC) and structural buckling^10^. In addition, the band gap can be tuned by an electric field along the buckling direction^11^,^12^. Silicene would be an excellent material for electronic applications due to its compatibility with the existing Si-based technology. Indeed, both silicene and germanene have been grown on gold and silver surfaces at room temperature^13^,^14^,^15^, and silicene field effect transistors have been demonstrated^16^. Theoretical studies have also predicted the stability of silicene on non-metallic substrates such as graphene^17^, boron nitride, and silicon carbide^18^.

Effects of circularly polarized light on silicene have been studied in ref. 9. In the present work, we address the Floquet edge states induced by circularly, linearly, and elliptically polarized light in germanene nanoribbons and the corresponding transport properties. We demonstrate that, by breaking the time reversal symmetry, it is possible to achieve full valley polarization because of an unbalanced number of counter-propagating chiral edge channels associated with the two valleys in the high frequency regime. Nontrivial edge states lead to a quantized Hall effect. The band structure can be tuned by means of the competition between the light and a uniform external electric field applied along the buckling direction. Analysis of the transport properties confirms a nontrivial nature of the edge states under circularly and elliptically polarized light.

## Results

We consider Dirac particles in a buckled honeycomb lattice, coupled to an in-plane time-dependent and spatially homogeneous vector potential *A*(*τ*) of period *T* = 2*π*/Ω, Ω being the frequency of the polarized light, and use the Floquet formalism. In general, the hopping parameters to the *j* nearest neighbors in the presence of polarized light read 
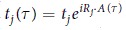
, where *t*_*j*_ is the hopping parameter without light. The vector potential has the form 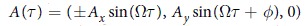, with the phase difference *ϕ* taking into account the in-plane rotation of the light, which is zero for linear, *π*/4 for elliptical, and *π*/2 for circular polarization. The +/− sign refers to right/left circular polarization and *R*_*j*_ is the lattice vector to neighbor *j*. Due to the time and spatial periodicity, the system is described by Floquet-Bloch states, which fulfill the Floquet eigenvalue problem. To study the band structure, we adopt a two band tight binding model. Including only nearest neighbor hopping is sufficient to capture the band edge properties of both valleys. Extending the Hamiltonian of refs 9 and 11 by including polarized light, without considering the spin degree of freedom, the tight binding Hamiltonian can be written as a 2 × 2 matrix in momentum space


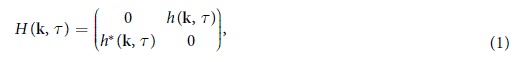


where 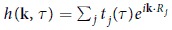. Assuming that the hopping parameter is the same for all nearest neighbors, we have for germanene *t* = 1.3 eV^11^ and *R*_1_ = *a*(1, 0), 
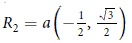
, 
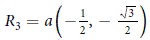
 with the in-plane nearest neighbor distance *a*. The Fourier transformed time-dependent hopping term reads


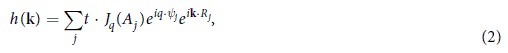


where *A*_1_ = *aA*_*x*_, 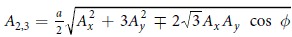, *ψ*_1_ = 0, 
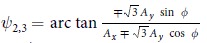
 and *J*_*q*_ is the *q*th Bessel function of the first kind. In the following we adopt *A*_*x*_ = *A*_*y*_ = *A*.

The SOC is stronger in silicene and germanene than in graphene because Si and Ge atoms are heavier than C atoms. Considering only the on-site contribution of the SOC, the full tight binding Hamiltonian reads


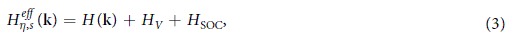


with *H*_SOC_ = *λsησ*_*z*_ (*λ* characterizes the strength of the SOC) and *H*_*V*_ = *λ*_*V*_*σ*_*z*_ (*λ*_*V*_ = 2*lE*_*z*_ is the staggered sublattice potential generated by the electric field *E*_*z*_ along the buckling direction when the two sublattices are separated by a distance of 2*l*). For germanene we have *l* = 0.3 Å^11^. The real spin of the Dirac fermions is denoted by *s* = ±1 and the valleys are represented by *η* = ±1.

**Figure 1:**
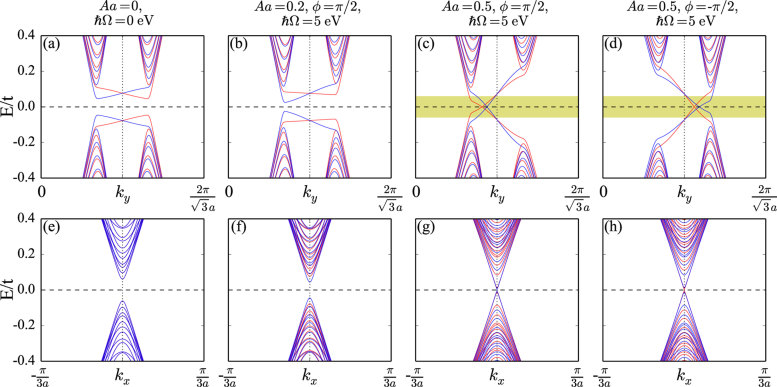
Band structures of germanene zigzag (**a**–**d**) and armchair (**e**–**h**) nanoribbons in an electric field given by *λ*_*V*_ = 0.1 eV, with and without circularly polarized light.

Circularly polarized light in the high frequency regime usually yields a Haldane gap by time reversal symmetry breaking^19^, encoded as mass term of opposite sign for the two Dirac cones. This is consistent with previous studies working within the single Dirac cone approximation and restricted to a weak driving field^2^,^9^. It follows from this mechanism that a change in the chirality of the field changes the sign of the mass term. We study the band structures of germanene nanoribbons in an static electric field, without and with circularly polarized light. Without light, see Fig. 1(a,e), a band gap is opened by the electric field, which breaks the inversion symmetry. For zigzag nanoribbons we obtain a large spin splitting at 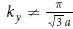, especially at the two K valleys, while for armchair nanoribbons both K points are projected to *k*_*x*_ = 0. The spin up and down bands are degenerate due to the additional mirror symmetry. When the circularly polarized light is turned on, see Fig. 1(b–d,f–h), the band gap decreases at one valley and increases at the other, reflecting opposite signs of the effective mass term. Switching the chirality of the light from right to left-handed changes the sign of the mass term, which is demonstrated in Fig. 1(c,d). When the amplitude of the light grows from *Aa* = 0.2 in Fig. 1(b,f) to *Aa* = 0.5 in Fig. 1(c,g) the band gap closes with nontrivial edge states, which indicates a topological phase transition. The yellow shaded area is the energy range that is covered only by nontrivial edge states.

**Figure 2:**
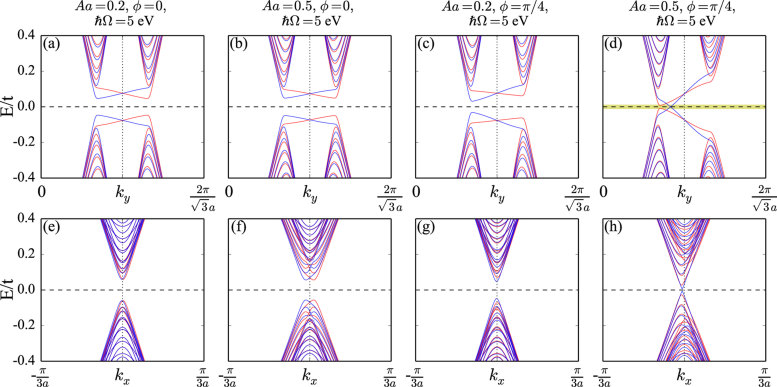
Band structures of germanene zigzag (**a**–**d**) and armchair (**e**–**h**) nanoribbons in an electric field given by *λ*_*V*_ = 0.1 eV, with linearly and elliptically polarized light.

For linearly polarized light we have *ψ*_1,2,3_ = 0, which means that the renormalized hopping integrals are real numbers. The time reversal symmetry is reserved, as indicated by the bands of the germanene nanoribbons in Fig. 2(a,b,e,f). The linearly polarized light only induces small anisotropic hopping components, which makes the bands similar to those without light, see Fig. 1(a,e). No edge states are found inside the band gap, which means that the system is a trivial insulator. The difference to circularly polarized light is that for the armchair nanoribbons, see Fig. 2(b), the spin up and down bands are split in momentum, as the anisotropic hopping breaks the inversion symmetry. For elliptically polarized light (*ϕ* = *π*/4) the time reversal symmetry is broken, see Fig. 2(c,d,g,h), and valley polarization appears, similar to the circularly polarized light. Indeed, when moving the phase of the light from circular to elliptical, the valley polarization is suppressed but the edge states survive. Armchair nanoribbons show the interesting feature that the band minima and maxima are slightly displaced from *k*_*x*_ = 0.

We next study the effect of circularly polarized light in the high frequency regime within the *k* · *p* model. We describe germanene by an Hamiltonian in the *xy*-plane,


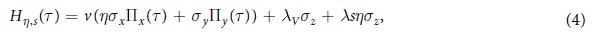


where (*σ*_*x*_, *σ*_*y*_, *σ*_*z*_) is the vector of Pauli matrices and *v* denotes the Fermi velocity of the Dirac fermions. In our notation the spin quantization axis is chosen along the *z*-direction. We use the gauge in the two-dimensional canonical momentum **Π**(*τ*) = **P** − *e***A**(*τ*) with the vector potential **A**(*τ*) = (±*A* sin Ω*τ*, *A* cos Ω*τ*), where *A* = *E*/Ω with *E* being the amplitude of the electric field **E**(*τ*) = ∂**A**(*τ*)/∂*τ*. The gauge potential satisfies time periodicity *A*(*τ* + *T*) = *A*(*τ*) with *T* = 2*π*/Ω. As long as the photon energy is much larger than the kinetic energy of the electrons, *H*_*η*,*s*_(*τ*) can be reduced to an effective *static* (time-independent) Hamiltonian 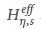 using Floquet theory^2^, which gives results in excellent agreement with experiments^4^. 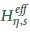 is defined through the time evolution over one period, 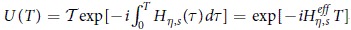, where 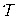 is the time ordering operator. Using perturbation theory and expanding *U*(*T*) in the limit of large Ω, we obtain


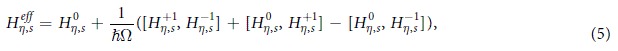


where 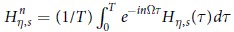 is the *n*th Fourier harmonic of the time-periodic Hamiltonian. Notice that Eq. (5) is only valid for 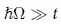 with 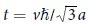 and *a* = 2.348 Å^9^. Indeed, for 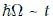 multiple photon absorption/emission processes must be accounted for, which implies that higher orders in the expansion of *U*(*T*) should be retained. On the other hand, the condition 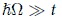 (*t* is proportional to the bandwidth) can be achieved experimentally^4^,^20^. We focus on the impact of high frequency light on the low energy bands and assume that any direct optical process involving high energy bands only weakly affects the low energy band structure. Still, due to the presence of these high energy processes, the effective power of the incident light is reduced.

Applying Eq. (5), Eq. (4) yields


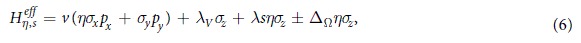


where 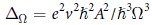 is the effective energy term describing the effects of the circularly polarized light, which essentially renormalizes the mass of the Dirac fermions. For right circular polarization the band gap is increased in the *K* valley and reduced in the *K*′ valley, whereas for left circular polarization the effect is reversed. After diagonalization we obtain the eigenvalues


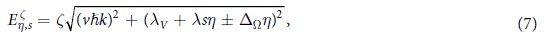


where *ζ* = ±1 represents the conduction and valence bands, respectively. The impact of high frequency light on the band structure is illustrated in Fig. 1 for *λ*_*V*_ = 0.1 eV and *λ* = 0.043 eV^9^. We set *ħ*Ω = 5 eV, which corresponds to a band gap variation of Δ_Ω_ = 0.05 eV for *evA* = 0.5 eV. Such a large value of *ħ*Ω ensures that the low energy bands are only affected by virtual emission/absorption processes, while higher energy processes only affect the effective power of the incident light, see also refs 2,7,9,20 and 21. The energy correction Δ_Ω_ can be tuned by varying the amplitude of the light or electric field.

We turn to the properties of the different edge states appearing in our system. In the high frequency regime the Floquet sidebands are well separated from each other. Thus, all topological properties can be studied within a two-band approximation and the zero energy modes behave equivalently to those of static systems. This demonstrates how it is possible to manipulate the two valleys by just tuning the chirality and frequency of the light. Edge states in only one valley confirm the valley imbalance in the high frequency regime, see Fig. 1(c,d). Two aspects are worth noticing: First, since right-handed circular polarization enhances the band gap for the *K* valley and reduces it for the *K*′ valley (left-handed circular polarization has the opposite effect), only *one* valley (here *η* = −1) is relevant for the low-energy electronic properties. Second, we obtain spin and valley polarized edge states in one of the two valleys, while in the absence of high frequency light the edge states persist in both valleys, as shown in Fig. 1(a). Nevertheless, due to the fact that there is an imbalance of the two valleys (by the combination of light and an external perpendicular electric field) it is possible to obtain *fully* spin-polarized transport by tuning the Fermi level. Since the system is fully valley-polarized, only one of the two valleys contributes to the transport.

To investigate the effect of polarized light on the transport properties, we further study the conductance of zigzag nanoribbons of 135 Å width, focusing on the high frequency regime. A central scattering region of 244 Å length (60 unit cells) is considered. The transmission coefficient is calculated using the generalized Fisher-Lee relation^2^,^22^


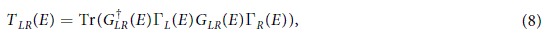


where Γ_*L*(*R*)_(*E*) represents the coupling between the scattering region and the left(right) reservoir. Moreover, *G*_*LR*_(*E*) is the Floquet Green’s function^2^,^23^,^24^, which is calculated by a recursive algorithm. To obtain the quantized conductance in the nontrivial band gap, we sum over all sidebands. This means all virtual absorption and emission processes in both reservoirs and the scattering region are taken into consideration.

**Figure 3:**
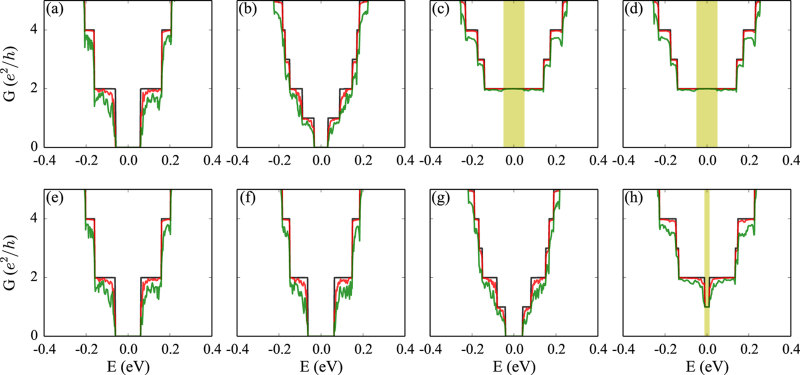
Conductance of germanene zigzag nanoribbons. The parameters in (**a**–**d**) are the same as in Fig. 1(a–d) and those in (**e**–**h**) are the same as in Fig. 2(a–d). The black, red, and green lines represent results for the clean system and for systems with disorder strengths of *W* = 0.13 and 0.39 eV, respectively.

Without light, see Fig. 3(a), the band gap opening due to the static electric field leads to zero conductance around the Fermi level. For circular polarized light of small amplitude, see Fig. 3(b), only one of the two valleys contributes to the transport, as the time reversal symmetry is broken. When the amplitude is sufficient to close the band gap and reopen a nontrivial band gap, see Fig. 3(c,d), the spin polarized edge states cause a quantized conductance in the band gap. Disorder is simulated by an additional random on-site energy, which is evenly distributed between −*W*/2 and *W*/2, with *W* being the disorder strength. According to Fig. 3(a,b), disorder strongly suppresses the conductance around the Fermi level, since the edge states contributing to the transport are trivial. On the other hand, in the case of nontrivial edge states, see Fig. 3(c,d), the quantized conductance around the Fermi level is almost not affected by the disorder. It is also found that right and left-handed light leads to the same transport properties. For linear polarized light, see Fig. 3(e,f), the results are similar to case without light, since the hopping is anisotropic. For elliptically polarized light of small amplitude, see Fig. 3(g), the conductance is suppressed by disorder. A growing amplitude, see Fig. 3(h), results in a small nontrivial band gap so that the edge states are again immune to disorder, which is reflected by a quantized conductance in the band gap.

In general, it is interesting how an electronic system in thermal equilibrium with Floquet states can be achieved with a topological band structure. We explicitly demonstrate that for high frequency light, where the electrons cannot directly absorb photons, the transport properties of the nonequilibrium system are well approximated by the static effective Hamiltonian that incorporates the virtual photon absorption processes. In particular, the occupations of the states are close to the filling of the photon-dressed bands. Without high frequency light the system is in a low temperature ground state with a chemical potential, where all the electrons are essentially in energy eigenstates. According to the adiabatic theorem for periodically driven systems^2^, energy eigenstates can be modified into Floquet states by adiabatic application of the driving light. We note that the time scale required to be approximately adiabatic is surprisingly short but was fulfilled in recent experiments^4^,^5^,^6^. Due to the topological nature, the described effects should generally be stable against sample imperfections.

## Discussion

We propose to use a static electric field together with circularly, linearly, or elliptically polarized light for tuning the band structure of germanene and enabling valley-polarized nanoelectronics. All our findings for germanene also apply to silicene. We have demonstrated that under circularly polarized light the band gaps of the *K* and *K*′ valleys are modified in opposite ways, leading to full valley polarization. This phenomenon leads to remarkable effects, such as the emergence of Hall plateaus, accompanied by an increase in the spin polarization of the flowing electrons. It is possible to control the charge transport in opposite valleys by changing the polarity of the light. Our predictions can be realized experimentally by the setup used in refs 4, 5, 6 for topological insulators. The effects found for elliptically polarized light are similar. For linearly polarized light the spin-split edge states are paired due to the presence of time reversal symmetry. The calculated transport properties show that the conductance contribution of trivial edge states is strongly suppressed by disorder, in contrast to nontrivial edge states, reflecting their topological nature. The discussed results open promising opportunities for the design of tunable spintronic and valleytronic devices.

## Additional Information

**How to cite this article**: Tahir, M. *et al*. Floquet edge states in germanene nanoribbons. *Sci. Rep.* **6**, 31821; doi: 10.1038/srep31821 (2016).
